# Trends in inequalities in child stunting in South Asia

**DOI:** 10.1111/mcn.12517

**Published:** 2017-10-19

**Authors:** Aditi Krishna, Iván Mejía‐Guevara, Mark McGovern, Víctor M. Aguayo, S. V. Subramanian

**Affiliations:** ^1^ Iris Group International Chapel Hill North Carolina USA; ^2^ Department of Biology Stanford University Stanford California USA; ^3^ Stanford Center for Population Health Sciences Stanford University School of Medicine Palo Alto California USA; ^4^ CHARMS ‐ Centre for Health Research at the Management School Queen's University Belfast Belfast UK; ^5^ UKCRC Centre of Excellence for Public Health (Northern Ireland) Belfast UK; ^6^ UNICEF, South Asia Kathmandu Nepal; ^7^ Harvard T.H. Chan School of Public Health Boston Massachusetts USA; ^8^ Harvard University Center for Population and Development Studies Cambridge Massachusetts USA

**Keywords:** health inequalities, social determinants of health, social factors, South Asia, stunting, undernutrition

## Abstract

We analysed socio‐economic inequalities in stunting in South Asia and investigated disparities associated with factors at the individual, caregiver, and household levels (poor dietary diversity, low maternal education, and household poverty). We used time‐series analysis of data from 55,459 children ages 6–23 months from Demographic and Health Surveys in Bangladesh, India, Nepal, and Pakistan (1991–2014). Logistic regression models, adjusted for age, sex, birth order, and place of residency, examined associations between stunting and multiple types of socio‐economic disadvantage. All countries had high stunting rates. Bangladesh and Nepal recorded the largest reductions—2.9 and 4.1 percentage points per year, respectively—compared to 1.3 and 0.6 percentage points in India and Pakistan, respectively. Socio‐economic adversity was associated with increased risk of stunting, regardless of disadvantage type. Poor children with inadequate diets and with poorly educated mothers experienced greater risk of stunting. Although stunting rates declined in the most deprived groups, socio‐economic differences were largely preserved over time and in some cases worsened, namely, between wealth quintiles. The disproportionate burden of stunting experienced by the most disadvantaged children and the worsening inequalities between socio‐economic groups are of concern in countries with substantial stunting burdens. Closing the gap between best and worst performing countries, and between most and least disadvantaged groups within countries, would yield substantial improvements in stunting rates in South Asia. To do so, greater attention needs to be paid to addressing the social, economic, and political drivers of stunting with targeted efforts towards the populations experiencing the greatest disadvantage and child growth faltering.

## 1 INTRODUCTION

Nearly one in four children under 5 years worldwide is stunted (UNICEF, 2016). Stunting, measuring chronic nutritional deficiency, is marked by being two standard deviations (SDs) below the median height‐for‐age *z*‐scores (HAZ) using World Health Organization (WHO) Multicentre Growth Reference Standards (WHO, 2006). Nearly 50% of stunted children live in Bangladesh, India, and Pakistan (UNICEF, 2016). Stunting declined globally by 40% over the past 2 decades; however, 37% of children under 5 years living in South Asia continue to experience stunted growth with persistently higher rates among poor children from rural areas (UNICEF, 2013, 2016).

Much evidence suggests that stunted children suffer from worse health (R. E. Black, Alderman, et al., 2013) and poorer developmental outcomes (M. M. Black et al., 2016). Recognizing the importance of early child nutrition, much global attention has focused on averting child undernutrition (*Scaling Up Nutrition: A Framework for Action*, 2010; UN, 2015; United Nations, 2015). One key recommendation from these initiatives is the need for better evidence regarding who is most vulnerable to stunting (R. E. Black, Alderman, et al., 2013).

Following on from this recommendation, our analysis seeks to provide an assessment of patterns in child stunting in South Asia and the extent of socio‐economic inequality in this outcome. We analyse the prevalence of child stunting in Bangladesh, India, Nepal, and Pakistan, selecting these countries because they bear 95% of South Asia's stunting burden (UNICEF, 2013, 2016). Our study examines the patterning of child stunting along three dimensions of disadvantage: (a) children's access to food, represented by dietary diversity; (b) social, represented by maternal educational attainment; and (c) economic, represented by household wealth. Together, these three types of socio‐economic disadvantage represent three, nested levels of the determinants of child nutrition—child‐level, mother‐level, and household‐level—in UNICEF's conceptual model of nutrition (UNICEF, 2013). Although much work considers socio‐economic gradients in child stunting (Akhtar, 2015; Di Cesare et al., 2015; Gaiha & Kulkarni, 2005; Headey, 2013; Headey & Hoddinott, 2014; Headey, Hoddinott, & Park, 2016; Kanjilal, Mazumdar, Mukherjee, & Rahman, 2010; Kumar & Kumari, 2014; Kumar, Kumari, & Singh, 2014; Menon, 2012), to our knowledge, only a few studies consider all three types of disadvantage in South Asia (Di Cesare et al., 2015; Fenske, Burns, Hothorn, & Rehfuess, 2013; Gaiha & Kulkarni, 2005). We concentrate on these three factors at the individual, caregiver, and household levels, a focus which reflects our interest in the impact of socio‐economic disadvantage on stunting. Although an assessment of all determinants is beyond the scope of this analysis, this does not imply that other risk factors are not important. Nevertheless, among the multiplicity of risk factors for child stunting, poor dietary diversity, low levels of maternal education, and household poverty were three of five determinants—the other two being short maternal stature and maternal underweight—that had the greatest relative contribution to stunting risk (Corsi, Mejía‐Guevara, & Subramanian, 2016). Our work extends evidence from country‐specific studies by comparing socio‐economic patterns in four South Asian countries, updating older analyses with more recent data, and providing further analysis of key underlying factors associated with child stunting. We investigate absolute and relative inequalities between groups characterized by these three dimensions of socio‐economic disadvantage in UNICEF's conceptual model of nutrition and examine how these disparities change over time. Our findings seek to inform future efforts to avert child stunting in the groups that are most vulnerable. On the basis of the existing literature, we hypothesize that socio‐economic disadvantage is an important determinant of stunting at each of the levels we consider: individual, caregiver, and household.

**Key messages**

- A comprehensive assessment of stunting in South Asia reveals substantial differences in the performance of countries in reducing the prevalence of undernutrition, with Nepal achieving the greatest declines and Pakistan the least.
- Socio‐economic adversity at the individual, caregiver, and household level was associated with increased risk of stunting.
- Stunting is concentrated among households experiencing multiple types of disadvantage (poor dietary diversity, low levels of maternal education, and household poverty).
- Inequalities in stunting rates have been maintained due to greater relative declines in certain socio‐economic groups despite overall declines in stunting rates in all countries.

## 2 METHODS

### 2.1 Data

We used data from 15 Demographic and Health Surveys (DHS; Corsi, Neuman, Finlay, & Subramanian, 2012) from Bangladesh, India, Nepal, and Pakistan between 1991 and 2014. Further information including details of the sampling design and weights is provided in Supporting Information.

The original sample size is composed of 75,454 children ages 6–23 months. Our study focuses on children ages 6–23 months because the first 2 years of life are critical for growth faltering (Victora, de Onis, Hallal, Blossner, & Shrimpton, 2010), and dietary diversity is typically assessed among children older than 6 months, who are no longer exclusively breastfed (WHO, UNICEF, USAID, AED, UCDavis, IFPRI, 2008a, 2008b). Because information on dietary diversity is not available prior to 6 months, we are unable to include infants under this age. We also excluded 4,460 children who died before the time of the interview, 835 multiple births, 8,835 lacking anthropometric measures, 5,821 children with biologically implausible height values (using the standard cut‐off of ≤ or ≥6 *SD*; WHO, 2006), and 44 observations with missing data on mother's education. Our final analytic sample was 55,459 children. In dietary diversity analyses for India and Pakistan, only children from the most recent survey years were included due to the lack of availability and quality of food‐related variables and data on feeding practices in the earliest survey years (*n* = 13,570). For Bangladesh and Nepal, we were able to use data on dietary diversity from the earliest and most recent surveys (*n* = 6,284; Table S1).

### 2.2 Outcome

Linear growth is measured as length using Shorr measuring boards, which are adjustable to the nearest millimetre (WHO, 2006). Measurements were standardized to height‐for‐age *z*‐scores using age‐ and sex‐specific growth standards from the WHO (WHO, 2006). The key outcome of interest—stunting—was estimated using the command zscore06 available in Stata (Leroy, 2011), designed to consistently estimate standardized anthropometric measures using these standards.

### 2.3 Explanatory variables

We considered three types of disadvantage as explanatory variables operating at three different levels—dietary diversity at the child level, maternal education at the caregiver level, and poverty at the household level. These three nested levels of determinants reflect those proposed by the UNICEF conceptual model of nutrition. Dietary diversity is measured as a summative score of 24‐hr recall of the following seven food groups described in the guidelines for assessing infant and young child feeding (Corsi et al., 2016; WHO, UNICEF, USAID, AED, UCDavis, IFPRI, 2008b): (a) grains, roots, and tubers; (b) legumes and nuts; (c) dairy products (milk, yogurt, and cheese); (d) flesh foods (meat, fish, poultry, and liver/organ meats); (e) eggs; (f) vitamin A rich fruits and vegetables; and (g) other foods and vegetables. Dietary diversity scores were categorized into three groups: low, medium, and high, depending on the level of dietary diversity and using the following cut‐offs from the dietary diversity score: 0–1, 2–3, and 4–7, respectively. The cut‐off 4–7 is defined as *minimum dietary diversity* for children ages 6–23 months (WHO, UNICEF, USAID, AED, UCDavis, IFPRI, 2008b). Mother's education was stratified according to three levels: no education, primary, and secondary and higher. Wealth quintiles were provided by the DHS using an asset‐based index (Rutstein & Johnson, 2004).

### 2.4 Analyses

We estimated the weighted prevalence of stunting over time and by dietary diversity group, mother's education level, and household wealth quintile. We estimated the percentage change in the prevalence of stunting between the earliest and latest estimates from each country within each group. We also estimated annualized changes in stunting prevalence for each subgroup to account for different time intervals between the earliest and latest surveys within countries. Annualized changes were calculated as average annual reduction rates (AARRs), as defined in the following expression (UNICEF, 2007)

(1) AARR=1−Yt+nYt1n,

where *Y*_*t*_ and *Y*_*t* + *n*_ are the prevalence of stunting at time *t* and *t + n*, respectively, and *n* is the number of years between *t* and *t + n*.

Absolute and relative differences were calculated between the two categories in groups with two categories and between the lowest and highest categories in groups with more than two categories, for both the earliest and latest survey years in every country. To take into account the complex design of the DHS survey (see Supporting Information for further details), confidence limits were approximated using the logit transformation procedure, which guarantees that the endpoints of the estimated proportion lie between 0 and 1 (Heeringa, West, & Berglund, 2010). Further, we assessed the relationship among the three types of socio‐economic disadvantage using the Spearman's rank correlation coefficient, for the earliest and latest survey years in the case of mother's education and household wealth, and the latest for children's dietary diversity (and earliest surveys for Bangladesh and Nepal).

We also used logistic regression models, adjusted for age, sex, birth order, and place of residency, to examine associations between stunting and multiple types of socio‐economic disadvantage. Analyses used pooled data with country‐fixed effects using information from the latest surveys in all four countries, as well as separate, country‐specific models. Additional models included interactions between different types of socio‐economic disadvantage to examine multiplicative effects of the three risk factors on stunting. We accounted for the complex survey design in our analyses, considering the two‐stage cluster design and sampling weights of the DHS surveys, as described in Supporting Information. All the calculations were performed using Stata 14 (StataCorp).

### 2.5 Ethics

Data from the DHS are anonymized and publicly available, rendering full ethics review unnecessary. The authors have received permission from the DHS programme to use data for this study.

## 3 RESULTS

### 3.1 Country analyses

Table 1 and Figure S1 present country‐level trends in child stunting. Approximately half of children were stunted at baseline in all countries. From the earliest DHS in the 1990s to the most recent surveys between 2006 and 2014, all countries experienced reductions in stunting prevalence with the largest declines recorded by Bangladesh and Nepal, where stunting prevalence declined annually by 2.9 percentage points and 4.1 percentage points, respectively, compared to 1.3 percentage points in India and 0.6 percentage points in Pakistan. At 43.5% (95% confidence interval, CI [42.3, 44.8]) and 39.7% (CI [35.4, 44.2]), India and Pakistan had the highest prevalence of stunting in the most recent DHS survey (2006 and 2013, respectively).

**Table 1:** Weighted prevalence (percentage) of stunting over time [95% confidence interval]

	DHS‐II	DHS‐III	DHS‐IV		DHS‐V	DHS‐VI	DHS‐VII	Change from the earliest to most recent year	% Reduction from earliest to most recent year	Average annual reduction rate (AARR)
Bangladesh (1997, 2000, 2004, 2007, 2011, 2014)		52.7 [49.6, 55.7]	45.1 [42.3, 48.0]	44.7 [41.9, 47.5]	36.0 [33.1, 39.0]	38.1 [35.6, 40.6]	32.0 [29.6, 34.4]	−20.7	39.3	2.9
India (1993, 1999, 2006)	51.3 [49.9, 52.6]		49.4 [48.2, 50.6]		43.5 [42.3, 44.8]			−7.8	15.2	1.3
Nepal (1996, 2001, 2006, 2011)		53.4 [50.7, 56.1]	47.2 [50.2, 44.1]		39.6 [43.5, 35.8]	28.3 [24.4, 32.6]		−25.1	47.0	4.1
Pakistan (1991, 2013)	45.2 [41.5, 49.0]					39.7 [35.4, 44.2]		−5.5	12.2	0.6

*Note*. In DHS‐II, “II” indicates that the DHS survey was conducted in the second phase. DHS = Demographic Health Survey.

### 3.2 Correlations among types of socio‐economic disadvantage

For all countries, we found statistically significant correlations between dietary diversity, mother's education, and household wealth (Table 2). In the most recent surveys, the Spearman's correlation between mother's education and wealth quintile was between 0.43 and 0.60 in all countries, with similar patterns in the earliest surveys, except for Nepal where the relationship was weaker (*r* = 0.33). Correlations between dietary diversity and mother's education varied in magnitude across all countries, ranging from 0.09 in Pakistan to 0.30 in Nepal in the most recent survey and from 0.14 in Nepal to 0.20 in Bangladesh in the earliest survey. For the correlation between dietary diversity and household wealth, correlations ranged from 0.13 in Bangladesh and Pakistan to 0.25 in India in the latest survey and from 0.13 in Nepal to 0.19 in Bangladesh in the earliest survey. Given these significant correlations, we conducted multicollinearity tests using the collin command in Stata. We found no evidence of multicollinearity either in the pooled data or country‐level samples in multivariable analyses that included all three levels of socio‐economic disadvantage.

**Table 2:** Pairwise Spearman's correlation^a^ between mother's education, wealth quintile, and dietary diversity score in the most recent and earliest surveys

		Bangladesh		India		Nepal		Pakistan	
		Mother's education	Wealth	Mother's education	Wealth	Mother's education	Wealth	Mother's education	Wealth
Most recent	Dietary diversity score	0.177	0.134	0.227	0.247	0.302	0.214	0.092	0.136
	Wealth	0.427		0.586		0.496		0.593	
Earliest	Dietary diversity score	0.203	0.194			0.136	0.126		
	Wealth	0.459		0.534		0.326		0.475	

*Note*. Due to data limitations, correlations with dietary diversity scores from India and Pakistan are available only for the latest survey years.

a All the correlations were statistically significant.

### 3.3 Trends in stunting by dietary diversity

**Figure 1:**
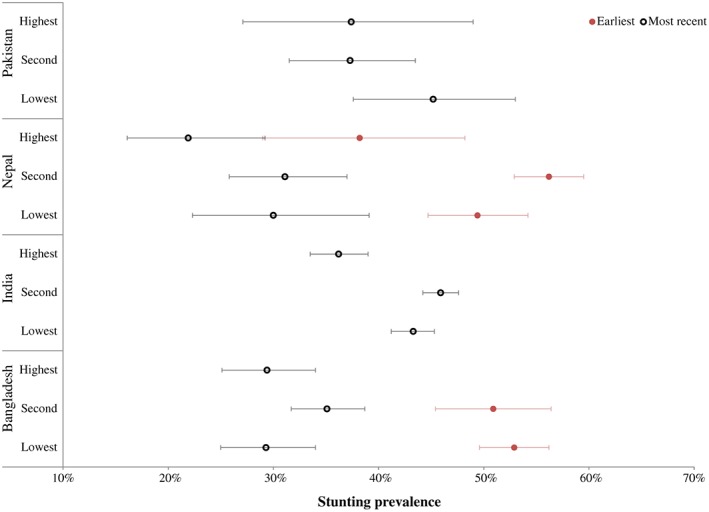
Stunting rates by dietary diversity score groups in the earliest and latest survey years. The points represent estimates and the error bars 95% confidence intervals. There is limited data for India and Pakistan

Children with the least diverse diet in the latest survey of India had significantly higher prevalence of stunting compared to children in the group with the most diverse diet (Figure 1 and Table 3). In Bangladesh and Nepal, a marked decrease in the prevalence of stunting was observed among the group with the least diverse diet between the earliest and most recent surveys, both experiencing a similar percent annual decline: the AARR was 3.4 in Bangladesh and 3.3 in Nepal. A similar reduction was also observed among the least deprived group in Nepal, with an AARR of 3.6.

**Table 3:** Weighted prevalence (percentage) of stunting, by dietary diversity score, in the most recent and earliest survey years^a^

Dietary diversity score, group	Bangladesh (2014, 1997)	India (2006, 1993)	Nepal (2011, 1996)	Pakistan (2013, 1991)
	% [95% CI]	% [95% CI]	% [95% CI]	% [95% CI]
Low				
Most recent	29.3 [25.0, 34.0]	43.3 [41.2, 45.3]	30.0 [22.3, 39.1]	45.2 [37.6, 53.0]
Earliest	52.9 [49.6, 56.2]		49.4 [44.7, 54.2]	
% Reduction	44.6		39.3	
AARR	3.4		3.3	
Medium				
Most recent	35.1 [31.7, 38.7]	45.9 [44.2, 47.6]	31.1 [25.8, 37.0]	37.3 [31.5, 43.5]
Earliest	50.9 [45.4, 56.4]		56.2 [52.9, 59.5]	
% Reduction	31.0		44.7	
AARR	2.2		3.9	
High				
Most recent	29.4 [25.1, 34.0]	36.2 [33.5, 39.0]	21.9 [16.1, 29.2]	37.4 [27.1, 49.0]
Earliest	NA		38.2 [29.0, 48.2]	
% Reduction			42.7	
AARR			3.6	
Between low‐/high	%	%	%	%
Absolute difference				
Most recent	−0.1	7.1	8.1	7.8
Earliest			11.2	
Relative difference				
Most recent	99.7	119.6	137.0	120.9
Earliest			129.3	

*Note*. “NA” indicates that we were unable to estimate the prevalence for that group. CI = confidence interval; AARR = average annual reduction rate.

a Due to data limitations, estimates from India and Pakistan are available only for the latest survey years.

### 3.4 Gradients in stunting by mother's education

**Figure 2:**
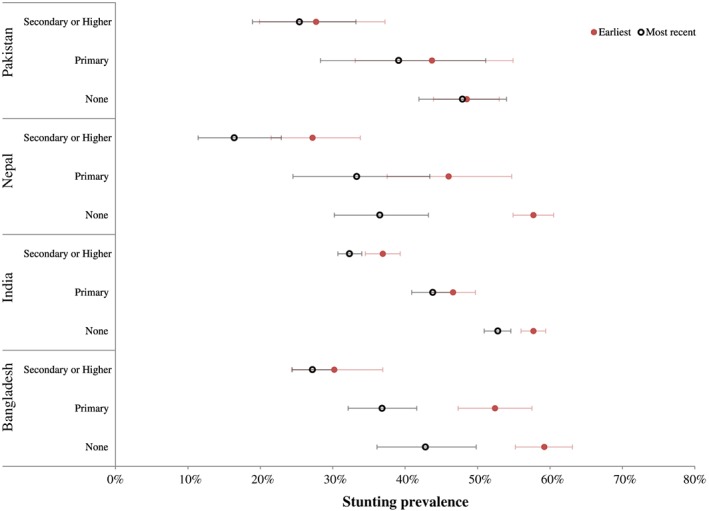
Stunting rates by mother's education in the earliest and latest survey years. The points represent estimates and the error bars 95% confidence intervals

There was lower stunting prevalence among children of more educated mothers (Figure 2 and Table 4). Stunting prevalence among children whose mothers had secondary education or higher was 27.2% (CI [24.4, 30.1]), 32.3% (CI [30.7, 34.0]), 16.4% (CI [11.4, 22.9]), and 25.4% (CI [18.9, 33.2]) in the most recent surveys in Bangladesh, India, Nepal, and Pakistan, respectively. In comparison, approximately 40–50% of children whose mothers were uneducated were stunted. All subgroups also experienced declines in all countries; however, there was some country variability in which education group experienced the largest reductions. In Bangladesh, there were similar stunting declines (AARRs of 1.9–2.1) among uneducated groups, and those with only primary education; in contrast, the more educated group experienced no significant annual decline in stunting. Meanwhile, in India and Nepal, the greatest reductions in stunting occurred in the uneducated group and in the group with secondary or more education. In Pakistan, no significant annual reductions were observed in any education group. Despite the varying rates of change among education groups, the education gradient in stunting was preserved in all four countries.

**Table 4:** Weighted prevalence (percentage) of stunting, by mother's education, in the most recent and earliest survey years

Mother's education	Bangladesh (2014, 1997)	India (2006, 1993)	Nepal (2011, 1996)	Pakistan (2013, 1991)
	% [95% CI]	% [95% CI]	% [95% CI]	% [95% CI]
None				
Most recent	42.8 [36.1, 49.8]	52.8 [50.9, 54.6]	36.5 [30.2, 43.2]	47.9 [41.9, 54.0]
Earliest	59.2 [55.2, 63.1]	57.7 [56.0, 59.4]	57.7 [54.9, 60.5]	48.5 [43.9, 53.0]
% Reduction	27.7	8.5	36.7	1.2
AARR	1.9	0.7	3.0	0.1
Primary				
Most recent	36.8 [32.1, 41.6]	43.8 [40.9, 46.7]	33.3 [24.5, 43.4]	39.1 [28.3, 51.1]
Earliest	52.4 [47.3, 57.5]	46.6 [43.6, 49.7]	46.0 [37.5, 54.7]	43.7 [33.1, 54.9]
% Reduction	29.8	6.0	27.6	10.5
AARR	2.1	0.5	2.1	0.5
Secondary or higher				
Most recent	27.2 [24.4, 30.1]	32.3 [30.7, 34.0]	16.4 [11.4, 22.9]	25.4 [18.9, 33.2]
Earliest	30.2 [24.3, 36.9]	36.9 [34.5, 39.3]	27.2 [21.5, 33.8]	27.7 [19.9, 37.2]
% Reduction	9.9	12.5	39.7	8.3
AARR	0.6	1.0	3.3	0.4
Between none and secondary+	%	%	%	%
Absolute difference				
Most recent	15.6	20.5	20.1	22.5
Earliest	29.0	20.8	41.3	20.8
Relative difference				
Most recent	157.4	163.5	222.6	188.6
Earliest	196.0	156.4	351.8	175.1

*Note*. CI = confidence interval; AARR = average annual reduction rate.

### 3.5 Patterning by household wealth

**Figure 3:**
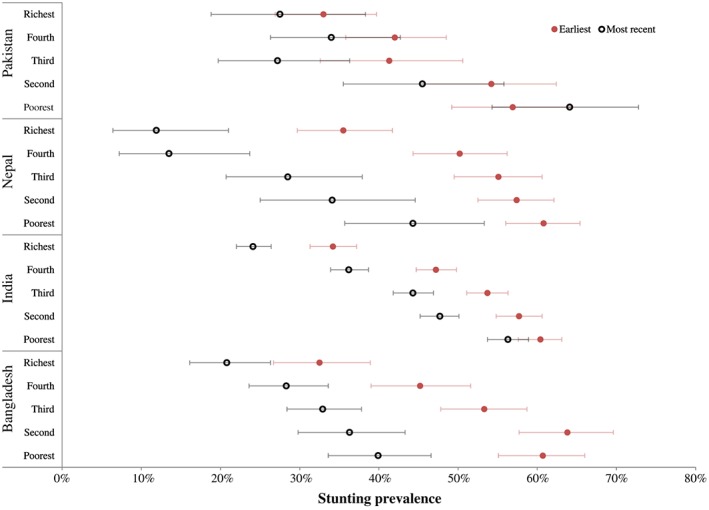
Stunting rates by household wealth quintile in the earliest and latest survey years. The points represent estimates and the error bars 95% confidence intervals

In both the earliest and latest surveys, there were higher stunting rates among children from poorer households with half to nearly two thirds of children in the lowest two quintiles experiencing stunting (Figure 3 and Table 5). Stunting rates declined for all quintiles in all countries; however, reductions were not uniformly distributed. In all four countries, the largest reductions in stunting prevalence occurred in the richest quintiles with the smallest declines in the poorest. For the richest quintile, stunting prevalence declined annually by 2.6, 2.7, 7.0, and 0.8 percentage points yearly in Bangladesh, India, Nepal, and Pakistan, respectively. In comparison, for the poorest quintile, stunting prevalence decreased by 2.4, 0.5, and 2.1 in Bangladesh, India, and Nepal, respectively, and increased by 0.5 percentage points in Pakistan. Absolute and relative differences between the poorest and richest quintiles increased in all four countries with the largest increases in Nepal and Pakistan.

**Table 5:** Weighted prevalence (percentage) of stunting, by wealth quintile, in the most recent and earliest survey years

Wealth quintile	Bangladesh (2014, 1997)	India (2006, 1993)	Nepal (2011, 1996)	Pakistan (2013, 1991)
	% [95% CI]	% [95% CI]	% [95% CI]	% [95% CI]
Poorest				
Most recent	39.9 [33.6, 46.6]	56.3 [53.7, 58.9]	44.3 [35.7, 53.3]	64.1 [54.3, 72.8]
Earliest	60.7 [55.1, 66.0]	60.4 [57.6, 63.1]	60.8 [56.0, 65.4]	56.9 [49.2, 64.2]
% Reduction	34.3	6.8	27.1	−12.7
AARR	2.4	0.5	2.1	−0.5
Poor				
Most recent	36.3 [29.8, 43.3]	47.7 [45.2, 50.1]	34.1 [25.0, 44.6]	45.5 [35.5, 55.8]
Earliest	63.8 [57.7, 69.6]	57.7 [54.8, 60.6]	57.4 [52.5, 62.1]	54.2 [45.7, 62.4]
% Reduction	43.1	17.3	40.6	16.1
AARR	3.3	1.5	3.4	0.8
Middle				
Most recent	32.9 [28.4, 37.8]	44.3 [41.8, 46.9]	28.5 (20.7, 37.9)	27.2 [19.7, 36.3]
Earliest	53.3 [47.8, 58.7]	53.7 [51.1, 56.3]	55.1 [49.5, 60.6]	41.3 [32.6, 50.6]
% Reduction	38.3	17.5	48.3	34.1
AARR	2.8	1.5	4.3	1.9
Rich				
Most recent	28.3 [23.6, 33.6]	36.2 [33.9, 38.7]	13.5 [7.2, 23.7]	34.0 [26.3, 42.7]
Earliest	45.2 [39.0, 51.6]	47.2 [44.7, 49.8]	50.2 [44.3, 56.2]	42.0 [35.8, 48.5]
% Reduction	37.4	23.3	73.1	19.0
AARR	2.7	2.0	8.4	1.0
Richest				
Most recent	20.8 [16.1, 26.3]	24.1 [22.0, 26.4]	11.9 [6.4, 21.0]	27.5 [18.8, 38.3]
Earliest	32.5 [26.7, 38.9]	34.2 [31.3, 37.2]	35.5 [29.7, 41.7]	33.0 [26.9, 39.7]
% Reduction	36.0	29.5	66.5	16.7
AARR	2.6	2.7	7.0	0.8
Rural‐/urban	%	%	%	%
Absolute difference				
Most recent	19.1	32.2	32.4	36.6
Earliest	28.2	26.2	25.3	23.9
Relative difference				
Most recent	191.8	233.6	372.3	233.1
Earliest	186.8	176.6	171.3	172.4

*Note*. CI = confidence interval; AARR = average annual reduction rate.

### 3.6 Association of stunting with different types of disadvantage

In separate country‐specific models, we observed mixed results when investigating the associations between stunting and children's dietary diversity, mother's education, and household wealth quintile (Table 6). For instance, in India, the risk of stunting for the most deprived groups was significantly higher than that for the least deprived groups in all types of disadvantage. Apart from India, Bangladesh was also the only country in which there were significant associations between maternal education and stunting. In terms of wealth, the risk of stunting was significantly higher for the poorest groups in all countries. Formal tests for heterogeneity in country‐specific results revealed that there was no heterogeneity for dietary diversity or maternal education; however, there was some evidence of effect modification for household wealth (see Table S2 and the corresponding paragraph).

**Table 6:** Odds ratio (OR) and 95% CI of stunting in adjusted models, pooled and country‐specific data in the latest survey year

	OR [95% confidence interval]					
	Pooled data	Pooled data—exclude India	Bangladesh 2014	India 2006	Nepal 2011	Pakistan 2013
Dietary diversity score, group						
Highest (ref)	1	1	1	1	1	1
Lowest	1.47	1.30	1.26	1.54	1.47	1.32
	[1.28–1.69]	[1.00–1.68]	[0.88–1.80]	[1.30–1.81]	[0.81–2.66]	[0.75–2.32]
Medium	1.35	1.17	1.29	1.42	1.18	0.87
	[1.20–1.52]	[0.95–1.46]	[1.00–1.67]	[1.23–1.63]	[0.73–1.90]	[0.49–1.53]
Mother's education						
Secondary or higher (ref)	1	1	1	1	1	1
None	1.51	1.62	1.79	1.48	1.31	1.79
	[1.34–1.69]	[1.25–2.09]	[1.25–2.58]	[1.30–1.68]	[0.69–2.51]	[0.99–3.24]
Primary	1.23	1.27	1.27	1.2	1.36	1.54
	[1.08–1.39]	[1.01–1.61]	[0.99–1.63]	[1.04–1.40]	[0.64–2.89]	[0.74–3.19]
Wealth, quintile						
Richest (ref)	1	1	1	1	1	1
Poorest	3.01	3.00	2.3	3.04	4.08	4.54
	[2.48–3.64]	[1.99–4.53]	[1.36–3.89]	[2.46–3.75]	[1.44–11.6]	[1.83–11.2]
Second	2.31	2.32	2.27	2.33	2.59	2.03
	[1.94–2.75]	[1.62–3.34]	[1.47–3.49]	[1.91–2.84]	[0.84–7.97]	[0.85–4.85]
Third	2.06	1.80	2.06	2.16	2.39	0.94
	[1.74–2.43]	[1.29–2.52]	[1.39–3.06]	[1.78–2.61]	[0.86–6.62]	[0.41–2.20]
Fourth	1.64	1.41	1.53	1.74	0.92	1.35
	[1.41–1.92]	[1.02–1.94]	[1.03–2.25]	[1.46–2.07]	[0.32–2.66]	[0.67–2.71]

*Note*. All models were additionally adjusted for child and household characteristics (age, sex, birth order, and place of residence) and country fixed‐effects.

After testing for heterogeneity, in fully adjusted models with pooled data, we found significant associations between stunting and children's dietary diversity, mother's education, and household wealth quintile (Table 6). The risks of stunting were significantly higher for children in the most disadvantaged groups; for example, a higher risk of stunting was observed for children in the group with the lowest dietary diversity (odds ratio [OR] = 1.47, 95% CI [1.28, 1.69]) compared to children in the group with the most diverse diet; for children of uneducated mothers (OR = 1.51, 95% CI [1.34, 1.69]) compared to children of the most educated mothers; and for children in the poorest wealth quintile (OR = 3.01, 95% CI [2.48, 3.64]) compared to children in the richest wealth quintile. In a model with interactions between dietary diversity and levels of mother's education, there were significant interactions (*p* value < .05) between these two types of socio‐economic disadvantage (Table S3). We conducted the same interaction tests in subsamples of the poorest and richest children (in separate models); however, we found no evidence of multiplicative effects of maternal education and dietary diversity among either the poorest or richest children. We also tested interactions between education and wealth and between dietary diversity and wealth in two separate models, but we found no evidence of multiplicative effects of the two types of disadvantage.

Differences in findings from pooled analyses, which showed distinct patterning in stunting rates along gradients of disadvantage, and country‐specific models, which had more inconsistent results, particularly for dietary diversity, can be attributed to the inclusion of India in the pooled analyses. However, the results for the most deprived groups remained consistent in the pooled analyses that excluded India, which was also consistent with the heterogeneity tests conducted earlier.

## 4 DISCUSSION

Our analysis of the prevalence and trends in child stunting in South Asia has three key findings. First, all countries had high stunting rates with nearly half of the children suffering from stunted growth at baseline. Stunting declined in all four countries with greater reductions in Bangladesh and Nepal. Second, adjusted models with pooled data showed higher rates of stunting among children who had poor diets, who had mothers with low educational attainment, or who lived in poor households. However, country‐specific analyses revealed varying vulnerability due to different dimensions of disadvantage. Third, the largest declines in stunting were seen in higher wealth quintiles, whereas the lower wealth quintiles recorded the smallest declines in stunting. Disparities in stunting rates were largely preserved over time, and in some cases worsened; for example, differences in stunting rates increased between children from poorer and richer households and between children whose mothers had low and high educational attainment.

South Asia bears a disproportionate burden of stunting: More than a third of children in the region are stunted compared to 10% in East Asia and the Pacific or 11% in Latin America and the Caribbean (UNICEF, 2016). Despite tremendous reductions in stunting from 61% to 37% from 1990 to 2016, four countries analysed in this study are among 21 countries with stunting rates above 40% (UNICEF, 2013). The significant burden of stunting in South Asia has been noted in the literature (Akhtar, 2015; R. E. Black et al., 2008; R. E. Black, Victora, et al., 2013; UNICEF, 2013) giving rise to the term “Asian enigma” (Ramalingaswami, Jonsson, & Rohde, 1996), which attributes markedly high and persistent stunting rates to gender inequalities. Substantial work has attempted to explore the causes of stunting, much of it linking stunting with single factors such as breastfeeding, micronutrient deficiencies, handwashing, and poor hygiene (studies are summarized in two recent reviews of the global evidence; Bhutta et al., 2008, 2013). A special issue in this Journal in 2016 focused on the particular causes and consequences of stunting in South Asia. Child feeding, women's nutrition, and household sanitation were posited as three critical determinants and points of intervention (Aguayo & Menon, 2016). Another article in the special issue attributed improvements in stunting to changes in material well‐being, female education, and sanitation (Headey et al., 2016). Our work is in line with these contributions, linking stunting with poor dietary diversity, low educational attainment among women, and household poverty.

Different rates of change in stunting prevalence between South Asian countries can be attributed to particular phenomena occurring within these countries. In Bangladesh, significant reductions in stunting have been linked to improvements in household economic status, increases in maternal and paternal education, greater availability and use of health services, better sanitation, reductions in fertility, increased agricultural productivity leading to greater food availability and security, and lastly, through specific improvements in nutrition such as a larger proportion of children being introduced to solid foods in timely fashion (Headey, Hoddinott, Ali, Tesfaye, & Dereje, 2014). Similar factors lead to improvements in child nutrition in Nepal (Headey & Hoddinott, 2014). Further comparison of DHS data showed that household wealth increased at a faster rate in Bangladesh and Nepal compared to India and Pakistan, suggesting that the greater increases in well‐being may explain higher reductions in stunting (Headey et al., 2016). In 2012, Nepal implemented a Multisector Nutrition Plan that integrates a nutritional interventions with water, sanitation, hygiene, social protection, and agriculture; although it is unlikely that the plan had any effect prior to its implementation, Nepal's efforts to improve child nutrition as a member of the Scaling Up Nutrition movement may explain the stunting declines in Nepal from 1996 to 2011 (Devkota, Adhikari, & Upreti, 2016). Bangladesh also adopted a new nutrition policy in 2016 (Ahmed, Hossain, Mahfuz, Choudhury, & Ahmed, 2016). Although these reasons may partially explain differing changes over time in stunting rates between the four South Asian countries, further research is needed.

Our findings on the experience of Bangladesh and Nepal, the increased risk associated with the predictors we demonstrate, and the distinct socio‐economic gradients in child stunting in South Asia, all further underscore the importance of social, economic, political, and environmental factors as the basic causes of child undernutrition. These factors drive the distribution of nutritional outcomes, namely, stunting, among children. The patterning we observed by mother's educational attainment as well as by household wealth quintile existed in all countries with markedly higher stunting rates among children with uneducated mothers and those residing in the poorest households. The role of maternal education and household wealth in explaining inequalities in child stunting has been well‐documented in the literature (Gaiha & Kulkarni, 2005; Kanjilal et al., 2010; Kumar & Kumari, 2014; Kumar & Singh, 2013; Menon, 2012). We also found asymmetric advances in the reduction in stunting along socio‐economic lines, particularly by wealth quintile. In concordance with similar findings in India (Kumar et al., 2014), our analysis also found greater reductions in stunting among wealthier groups. In Pakistan and India, the poorest quintile has experienced only single digit changes in stunting prevalence over time. In contrast, stunting prevalence in the wealthiest quintiles has declined by more than a third in both countries, giving rise to widening inequalities.

Overall, our analysis has the positive implication that there are likely to be opportunities to pursue further reductions in stunting. First, at the national level, the comparatively better performance of Bangladesh and Nepal indicates that it should be possible to achieve greater advances elsewhere in South Asia, and the experience of these countries will be of particular interest to India, Pakistan, and others that have yet to achieve similar improvements. In the above discussion, we have pointed to the literature that highlights potential causes of the relative success of Bangladesh and Nepal, and in the coming years, it will be of particular interest to examine how the recent adoption of nutrition strategies in these countries affects future improvements. Second, within countries, the fact that the greatest declines in stunting occurred among the wealthiest quintiles, and that these wealth gaps persisted over time, implies that extending the advantages of those who are better off to poorer children would result in further overall improvements. As well as being important from equity and economic standpoints, from the perspective of achieving reductions in stunting, elimination of socio‐economic inequality in child stunting by bringing prevalence among the worst off to match that among the best off in each country should therefore be a policy priority. It remains an open question as to how best to pursue this goal; however, doing so will likely require ensuring that the proceeds of economic growth are distributed equally among all households through targeting poverty reduction, improvements in diet and sanitation, and gender inequalities (McGovern, Krishna, Aguayo, & Subramanian, 2017).

### 4.1 Limitations

Although our study provides updated evidence from an in‐depth analysis of child stunting prevalence and trends in South Asian countries, with particular emphasis on differences in stunting rates between socio‐economic groups, there are a few limitations to our analysis. Data constraints were one example. The DHS surveys are implemented during different years, at different intervals, and with varying frequency. Our estimates take into account differences in time intervals between the earliest and latest surveys, but the prevalence is not comparable for the latest survey years when the timing of the last survey differs between countries. Also, we only had data for at least 2 years for only four of the most populous South Asian countries with the highest stunting prevalence—Bangladesh, India, Nepal, and Pakistan—rather than from the entire set of South Asian countries (WorldBank, n.d.). There was limited information available on dietary diversity in the early years for certain surveys (India and Pakistan). Furthermore, there are concerns using dietary diversity as a measure of nutritional adequacy; however, we were unable to supplement dietary diversity with other measures such as timely introduction of complementary foods, which is only available for children ages 6–8 months. We also considered adding in feeding frequency as another child‐level explanatory variable; however, we decided to exclude this indicator because in a previous study, we did not find a significant association between feeding frequency and stunting after controlling for sociodemographic characteristics and 11 additional risk factors for stunting (Kim, Mejía‐Guevara, Corsi, Aguayo, & Subramanian, 2017). Despite its limitations, we believe dietary diversity is the best measure of nutritional adequacy available in the DHS. Lastly, our analysis was limited in terms of the socio‐economic status variables with only education and wealth (measured through an asset‐based index; Filmer & Pritchett, 2001) available. Despite these shortcomings, largely related to data limitations, our analysis provides an updated analysis of child stunting in South Asia, highlighting marked inequalities in stunting rates.

## 5 CONCLUSION

The implications of there being nearly 80 million stunted children in South Asia, and the concentration of stunting prevalence among individuals facing one or more forms of socio‐economic disadvantage, are immense (UNICEF, 2016). Stunted children experience worse health outcomes, developmental deficits, and poorer livelihoods (R. E. Black, Alderman, et al., 2013; Grantham‐McGregor et al., 2007), leading to significant losses of human capital and lower productivity (Behrman, Bhalotra, Deolalikar, Laxminarayan, & Nandi, 2015; R. E. Black, Alderman, et al., 2013; Grantham‐McGregor et al., 2007; Horton & Steckel, 2011). From an equity perspective, the reciprocal nature of poverty and stunting is concerning as stunted individuals are excluded from participating in economic progress, compromising the notion of inclusive growth (Horton & Steckel, 2011).

High stunting rates in South Asia also challenge progress towards the sustainable development goals (SDGs), notably ending poverty in all its forms (SDG 1); promoting sustained, inclusive, and sustainable economic growth, full productive employment, and decent work for all (SDG 8); and reducing inequality within and between countries (SDG 10; UN, 2015). Indeed, the SDGs explicitly note the importance of nutrition with SDG 2 aiming to end hunger, achieve food security and improved nutrition, and promote sustainable agriculture and endorsing the WHO target to reduce the number of stunted children by 40% by 2025 (UN, 2015). Thus, increasing efforts to reduce child stunting in South Asia will move these countries closer towards achieving SDG 2, which will in turn contribute to achieving the broader SDG goals and targets related to child survival, growth, development, education, participation, and equity. Greater attention needs to be paid to addressing the social, economic, and political drivers of stunting, with targeted efforts towards the populations at a higher risk of persistent nutritional deprivation.

## CONFLICTS OF INTEREST

The authors declare that they have no conflicts of interest.

## CONTRIBUTIONS

AK, IM, MM, VA, and SVS conceptualized and designed the study. AK and IM carried out the initial analyses and drafted the initial manuscript. AK, IM, MM, VA, and SVS reviewed and revised the manuscript and approved the final manuscript as submitted.

## Supporting information


**Figure S1** Changes in stunting prevalence in South Asia, 1991–2014
**Table S1** Sample size by country, survey year, and three dimensions of deprivation (child dietary diversity, maternal education, and household wealth)
**Table S2** Test of heterogeneity (effect modification) for the association of stunting and dietary diversity score, mother's education, and wealth in unadjusted models using data for the latest survey year.
**Table S2** Odds Ratio (OR) and 95% CI of stunting in adjusted pooled models with Mother's education and dietary inadequacy interactions using data for the latest survey year
