# Supplementary material for: Trends in inequalities in child stunting in South Asia
Source: Matern Child Nutr. 2017 Oct 19;14(Suppl 4):e12517. doi: 10.1111/mcn.12517 (PMC6519254; doi:10.1111/mcn.12517)
Supplement: Supplementary file 1 — Figure S1 Changes in stunting prevalence in South Asia, 1991–2014 Table S1 Sample size by country, survey year, and three dimensions of deprivation (child dietary diversity, maternal education, and household wealth) Table S2 Test of heterogeneity (effect modification) for the association of stunting and dietary diversity score, mother's education, and wealth in unadjusted models using data for the latest survey year. Table S2 Odds Ratio (OR) and 95% CI of stunting in adjusted pooled models with Mother's education and dietary inadequacy interactions using data for the latest survey year [file MCN-14-e12517-s001.docx]

**Supplemental Material**

1. ***Overview of the Demographic and Health Surveys***

DHS sample surveys contain information on a variety of maternal and child health and nutrition indicators, including anthropometric, demographic and socioeconomic characteristics (1). Sampling weights are available in DHS to provide estimates that are representative at the country-, state-, and provincial levels (2).

DHS surveys are usually based on a stratified two-stage cluster design. Stratification is conducted by geographic location (usually urban or rural), and by states, provinces or geographical regions (depending on the country context). In the first stage of the sampling selection, primary sampling units (PSUs) are drawn within each stratum with probability proportional to size (PPS) sampling from a list of all enumeration areas which are non-overlapping, cover the entire country, and are taken from the latest population census (when available). In the second stage, households are drawn from every selected PSU with equal systematic probability from a list of all previously enumerated households (3).

**References**

1. Corsi DJ, Neuman M, Finlay JE, Subramanian SV. Demographic and Health Surveys: a profile. Int J Epidemiol. 2012;41(6):1602–13.

2. Measure DHS. Demographic and Health Surveys. Calverton, MD: DHS; 2009.

3. ICF International. *Demographic and Health Survey Sampling and Household Listing Manual*. Calverton, MD: Measure DHS, ICF International.

**Figure A1** Changes in stunting prevalence in South Asia, 1991-2014

**Table A1** Sample size by country, survey year, and three dimensions of deprivation (child dietary diversity, maternal education, and household wealth)

| **Country and survey year** | **Dietary Diversity Score Group** | | | **Wealth, quintile** | | | | | **Mother’s Education** | | |
| --- | --- | --- | --- | --- | --- | --- | --- | --- | --- | --- | --- |
|  | **Low** | **Medium** | **High** | **Poorest** | **Poor** | **Middle** | **Rich** | **Richest** | **None** | **Primary** | **Secondary or higher** |
| Bangladesh | 3,187 | 3,866 | 2,092 | 2,351 | 2,160 | 2,129 | 2,075 | 2,231 | 3,112 | 3,211 | 4,623 |
| 1997 | 1,079 | 369 | 0 | 329 | 339 | 277 | 278 | 250 | 808 | 425 | 240 |
| 2000 | 450 | 796 | 332 | 376 | 337 | 297 | 271 | 320 | 714 | 466 | 421 |
| 2004 |  |  |  | 397 | 313 | 336 | 294 | 392 | 565 | 535 | 632 |
| 2007 | 400 | 691 | 571 | 304 | 336 | 324 | 304 | 395 | 383 | 508 | 772 |
| 2011 | 669 | 1,001 | 559 | 467 | 426 | 434 | 455 | 455 | 353 | 660 | 1,224 |
| 2014 | 589 | 1,009 | 630 | 478 | 409 | 461 | 473 | 419 | 289 | 617 | 1,334 |
| India | 18,554 | 13,238 | 2,493 | 6,180 | 6,462 | 7,308 | 8,607 | 7,692 | 16,842 | 5,532 | 13,875 |
| 1993 | 9,204 | 0 | 0 | 1,782 | 1,820 | 2,020 | 2,716 | 2,413 | 6,011 | 1,662 | 3,078 |
| 1999 | 5,428 | 6,961 | 0 | 2,219 | 2,319 | 2,704 | 2,994 | 2,551 | 6,053 | 2,084 | 4,650 |
| 2006 | 3,922 | 6,277 | 2,493 | 2,179 | 2,323 | 2,584 | 2,897 | 2,728 | 4,778 | 1,786 | 6,147 |
| Nepal | 985 | 2,352 | 810 | 1,642 | 1,265 | 1,149 | 1,135 | 874 | 4,065 | 892 | 1,108 |
| 1996 | 599 | 1,211 | 121 | 515 | 402 | 376 | 394 | 289 | 1,574 | 203 | 199 |
| 2001 |  |  |  | 474 | 408 | 364 | 349 | 278 | 1,333 | 269 | 271 |
| 2006 | 255 | 802 | 482 | 453 | 324 | 280 | 270 | 212 | 879 | 295 | 365 |
| 2011 | 131 | 339 | 207 | 200 | 131 | 129 | 122 | 95 | 279 | 125 | 273 |
| Pakistan | 1,219 | 408 | 196 | 328 | 379 | 417 | 533 | 542 | 1,423 | 296 | 480 |
| 1991 | 945 | 0 | 0 | 156 | 201 | 243 | 344 | 377 | 974 | 147 | 200 |
| 2013 | 274 | 408 | 196 | 172 | 178 | 174 | 189 | 165 | 449 | 149 | 280 |
| Total | 23,945 | 19,864 | 5,591 | 10,501 | 10,266 | 11,003 | 12,350 | 11,339 | 25,442 | 9,931 | 20,086 |

**Table A2** Test of heterogeneity (effect modification) for the association of stunting and dietary diversity score, mother’s education, and wealth in unadjusted models using data for the latest survey year.

| **SES Variable** | **OR (95% CI)** | | | | | |
| --- | --- | --- | --- | --- | --- | --- |
|  | **Pooled data**  **(M-H)** | **Pooled data - exclude India (M-H)** | **Bangladesh 2014** | **India 2006** | **Nepal 2011** | **Pakistan 2013** |
| **Dietary Diversity Score,  tertile** |  |  |  |  |  |  |
| Highest (ref) | 1 | 1 | 1 | 1 | 1 | 1 |
| Lowest | 1.29 | 1.13 | 0.97 | 1.34 | 1.38 | 1.43 |
|  | (1.17 - 1.41) | (0.93 - 1.37) | (0.76 - 1.26) | (1.20 - 1.49) | (0.81 - 2.34) | (0.95 - 2.14) |
| Test of Homogeneity: Mantel-Haenszel (M-H) chi2(3) | 5.76 | 3.45 |  |  |  |  |
| Test that combined OR=1: M-H chi2(1) | 28.56** | 1.52 |  |  |  |  |
| **Mother’s Education** |  |  |  |  |  |  |
| Richest (ref) | 1 | 1 | 1 | 1 | 1 | 1 |
| Poorest | 2.44 | 2.69 | 2.31 | 2.39 | 3.23 | 2.95 |
|  | (2.26 – 2.62) | (2.24 – 3.23) | (1.76 – 3.04) | (2.21 – 2.59) | (2.14 – 4.92) | (2.08 – 4.19) |
| Test of Homogeneity: Mantel-Haenszel (M-H) chi2(3) | 3.53 | 2.35 |  |  |  |  |
| Test that combined OR=1: M-H chi2(1) | 588.92** | 115.88** |  |  |  |  |
| **Wealth, quintile** |  |  |  |  |  |  |
| Secondary or higher (ref) | 1 | 1 | 1 | 1 | 1 | 1 |
| None | 2.38 | 2.69 | 2.04 | 2.29 | 6.27 | 3.51 |
|  | (2.16 – 2.64) | (2.24 – 3.23) | (1.54 – 2.70) | (2.04 – 2.57) | (3.33 – 12.35) | (2.22 – 5.58) |
|  |  |  |  |  |  |  |
| Tests of Homogeneity: Mantel-Haenszel chi2 (3) | 14.49** | 12.89** |  |  |  |  |
| Test that combined OR=1 M-H chi2(1) | 291.74** | 88.97** |  |  |  |  |

Note: p-val < 0.05 (*), < 0.01 (**).

In separate unadjusted models for each domain of disadvantage, we formally tested for heterogeneity (or effect modification) of the odds ratios (ORs) for the most deprived groups (relative to the least deprived ones) in all countries. Results using Mantel-Haenszel (M-H) homogeneity tests revealed no significant evidence of heterogeneity across countries for groups with the least diverse diets (Mantel-Haenszel chi2(3) [M-H]: 5.76, p-val>0.05) and uneducated mothers (M-H: 3.53, p-val>0.05); however, we did find significant heterogeneity for the poorest groups (M-H: 14.49, p-val<0.05). This latter result may be due to the small sample size in Nepal among the richest groups (**Table A1**), probably reflected in the highest ORs observed among all countries and the fact that the heterogeneity test became not statistically significant after excluding Nepal. The heterogeneity test remained consistent after the exclusion of India, which accounts for nearly 70% of the total sample (**Table A1**), except for the analysis for dietary diversity, where the M-H test for the combined OR = 1 cannot be rejected after excluding India from the pooled sample.

**Table A2** Odds Ratio (OR) and 95% CI of stunting in adjusted pooled models with Mother’s education and dietary inadequacy interactions using data for the latest survey year

|  | **Stunting OR (95% CI)** | | |
| --- | --- | --- | --- |
| **SES variable** | **Pooled data** | **Only poorest** | **Only richest** |
| **Dietary Diversity Score** |  |  |  |
| Highest (ref) | 1 | 1 | 1 |
| Lowest | 1.62 | 1.31 | 1.85 |
|  | (1.33 - 1.97) | (0.53 - 3.24) | (1.27 - 2.70) |
| Medium | 1.61 | 1.5 | 1.25 |
|  | (1.37 - 1.89) | (0.70 - 3.24) | (0.95 - 1.64) |
| **Mother's education** |  |  |  |
| Secondary or Higher (ref) | 1 | 1 | 1 |
| None | 1.79 | 2.45 | 0.78 |
|  | (1.40 - 2.29) | (1.06 - 5.66) | (0.23 - 2.58) |
| Primary | 1.79 | 2.42 | 0.98 |
|  | (1.37 - 2.34) | (0.98 - 5.97) | (0.37 - 2.58) |
| **Interactions** |  |  |  |
| Education(Secondary or higher) X Dietary Inadequacy (highest) [ref] | 1 | 1 | 1 |
| None X Lowest | 0.89 | 0.92 | 1.35 |
|  | (0.66 - 1.19) | (0.33 - 2.51) | (0.31 - 5.88) |
| None X Medium | 0.77 | 0.73 | 2.37 |
|  | (0.58 - 1.01) | (0.30 - 1.78) | (0.64 - 8.76) |
| Primary X Lowest | 0.65 | 0.48 | 1.7 |
|  | (0.46 - 0.93) | (0.16 - 1.43) | (0.45 - 6.44) |
| Primary X Medium | 0.62 | 0.53 | 1.53 |
|  | (0.44 - 0.86) | (0.19 - 1.46) | (0.49 - 4.71) |
| Adjusted Wald Test for interaction | 2.89* | 0.86 | 1.02 |
| **Wealth, quintile** |  |  |  |
| Richest (ref) | 1 |  |  |
| Poorest | 2.99 |  |  |
|  | (2.47 - 3.62) |  |  |
| Second | 2.3 |  |  |
|  | (1.93 - 2.74) |  |  |
| Third | 2.04 |  |  |
|  | (1.73 - 2.41) |  |  |
| Fourth | 1.63 |  |  |
|  | (1.40 - 1.91) |  |  |
